# Supplementary material for: Causal interplay between lactose intolerance and gut microbiota: a combined bidirectional Mendelian randomization and in vivo validation study
Source: Front Nutr. 2026 Jun 1;13:1803337. doi: 10.3389/fnut.2026.1803337 (PMC13265576; doi:10.3389/fnut.2026.1803337)
Supplement: Supplementary file 1 [file Data_Sheet_1.zip › supplementary materials/Forward/leave one out cross validation/ebi-a-GCST90027774.finngen_R12_E4_LACTONAS.pdf]

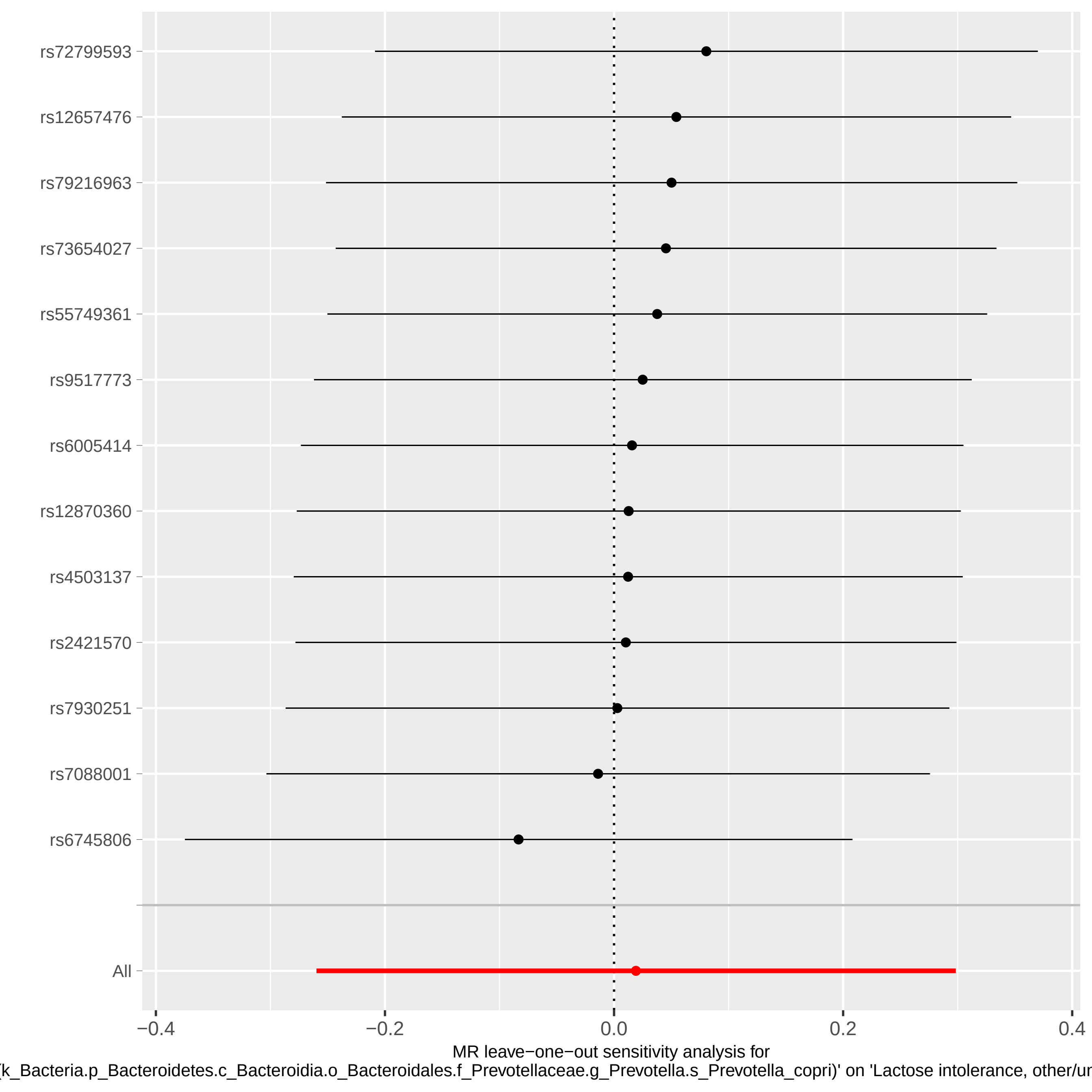

rs72799593
rs12657476
rs79216963
rs73654027
rs55749361
rs9517773
rs6005414
rs12870360
rs4503137
rs2421570
rs7930251
rs7088001
rs6745806
All
−0.4
−0.2
0.0
0.2
0.4
MR leave−one−out sensitivity analysis for
'(k_Bacteria.p_Bacteroidetes.c_Bacteroidia.o_Bacteroidales.f_Prevotellaceae.g_Prevotella.s_Prevotella_copri)' on 'Lactose intolerance, other/ur
